# Scintillating Grid Illusion Without the Grid

**DOI:** 10.1177/2041669520944418

**Published:** 2020-07-23

**Authors:** Toyomi Matsuno

**Affiliations:** Faculty of Economics, Hosei University, Tokyo, Japan

**Keywords:** visual illusion, scintillating grid illusion, surface filling-in, spatial vision

## Abstract

The scintillating grid illusion is a phenomenon where illusory black spots are perceived on white patches located at the intersections of a grid pattern. In this study, I report that the illusory spots as observed in the illusion are perceived with a stimulus pattern without grid bars. In two experiments, I investigated the perceptual properties of the scintillating illusion without grid bars. I found that the strength of the illusion depends on the contour shape of the patch components as in the scintillating grid illusion, while neither the density nor spatial alignment largely affect the illusory percepts. These findings undermine the previous theories on the mechanism of the scintillating grid illusion, as it was assumed that the grid bars are the essential component to induce the illusion. The results suggest that the illusory spots of the scintillating grid illusion could be induced by the limited processing of the patch stimuli in the peripheral vision and that the grid could play a supplementary role by enhancing the effect by further interfering with the processing.

Visual patterns that contain vertical and horizontal grid bars as components induce various illusory percepts (Hermann, 1870; Kawabe et al., 2010; Ninio & Stevens, 2000; Qian & Mitsudo, 2016). The scintillating grid illusion (Schrauf et al., 1997) is one prominent example, in which we perceive illusory black spots on white patches located at the intersections of the grid bars (Figure 1, left).

**Figure 1.**
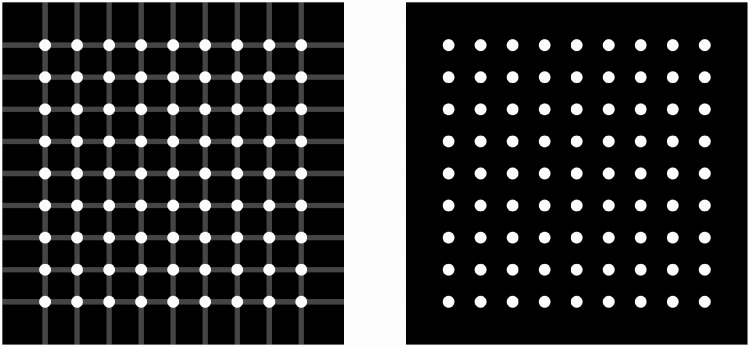
Scintillating Grid Illusion With and Without Grid.

Previous studies proposed some hypotheses on the underlying mechanism of the illusion, but they were inconclusive. Some suggested that the scintillating grid illusion could be a retinal phenomenon (Schrauf & Spillmann, 2000; Yu & Choe, 2006), while others stated that orientation processing of the grid bars in early visual cortices may play a critical role in inducing the illusory spots (Qian et al., 2012). These studies presupposed exclusive processes from each other, but they shared the view that the grid bars are the essential visual components that are directly involved in the generation of the illusory spots.

Here, I show that the illusory spots that are similar to those observed with the scintillating grid illusion are also perceived in the pattern without grid bars (Figure 1, right; see Supplemental Figure S1 for a more compelling example). The perceptual properties of the illusory spots are shared with those of the scintillating grid illusion, although the perceived strength of the illusion is much weaker. For example, as in the scintillating grid illusion, the illusory spots in the white patches can be perceived transiently, modulated depending on the eye movement and disappearing with fixation.

In this study, I investigated the perceptual characteristics of the illusion by varying the spatial properties of the stimulus. In Experiment 1, the effect of the stimulus shape and the density of the white patches were examined. In Experiment 2, the spatial alignment of the visual elements was varied. These experiments tested three issues. First, the illusion could depend on the contour shape of the patches, if the underlying mechanism of the illusion is shared with the scintillating grid illusion. A previous study on the scintillating grid illusion that examined the effect of the shape of the patches revealed that the illusion is much weaker with squared patches than with round ones (Qian et al., 2009). Second, the illusion could be weaker in sparse stimulus patterns, if the illusion was caused by local interference between adjacent visual elements. Previous studies of the scintillating grid illusion have proposed that the local spatial interaction in our visual processing could cause the illusory spots (e.g., Qian et al., 2009; Yu & Choe, 2006). Although the grid bars were absent from the stimulus, the local interference between contour lines of adjacent patches could play a similar role and induce the illusory spots. Third, the illusion could be stronger with aligned rather than misaligned patterns. The square lattice spatial arrangement of the visual elements could result in a similar output of the frequency- or orientation-selective spatial filtering to that with the stimuli with the orthogonal grid bars, and that could be essential for the illusion. In that case, misalignment of the patches could diminish the illusion.

## Experiment 1

In Experiment 1, the visibility of the illusion was examined by varying the shape of the patch contours and the stimulus density.

### Method

This and the following experiment were conducted in accordance with the Declaration of Helsinki, and the experimental designs were approved by the Research Ethics Committee of the Department of Economics at Hosei University (2019-S05). Written informed consent was obtained from each participant before conducting the experiments.

#### Participants

Twenty-eight observers (14 females and 14 males) ranging in age from 18 to 23 years (mean = 19.2, *SD* = 1.3) participated in Experiment 1. They were undergraduate students at Hosei University who participated for course credits. They were naïve as to the purpose of the experiments. All had normal or corrected-to-normal visual acuity.

#### Apparatus and Stimuli

The stimuli were generated on an Intel Core i7 computer and displayed on 23-in TFT monitors (EIZO, Foris FS2333) using custom-built software programmed in Visual C++. The stimulus images were displayed without antialiasing. The monitor resolution and refresh rate were set to be 1,920 × 1,080 pixels and 60 Hz, respectively. Participants observed the monitor at a viewing distance of approximately 50 cm using a chinrest and headrest in a dark experimental booth. Stimulus luminance was measured and adjusted daily using a colorimeter (Konica-Minolta, CS-100A). A computer mouse (Buffalo, BSMBU19BK) was used as a response device.

**Figure 2.**
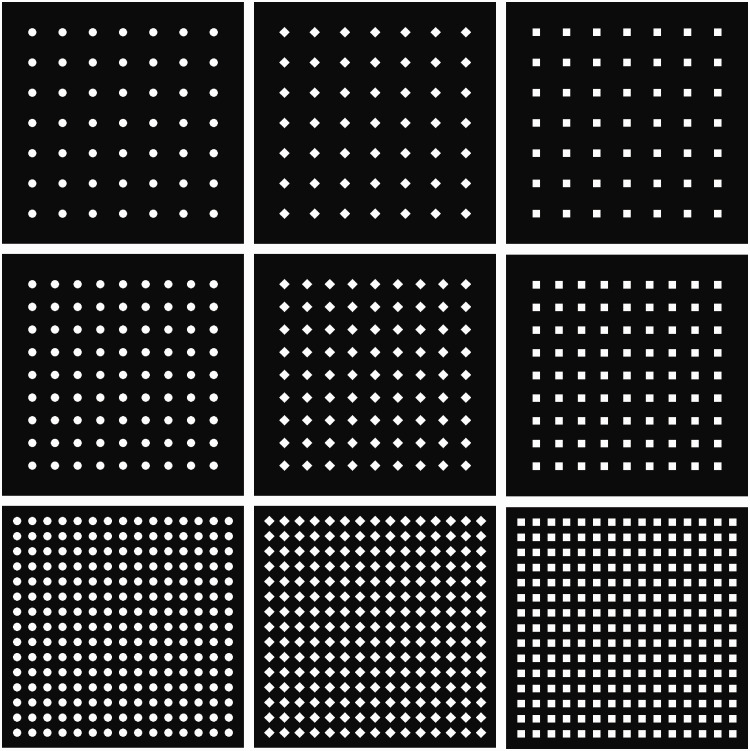
Stimuli Used in Experiment 1.

The properties of the stimuli were determined by referring to the stimuli properties used in the previous studies on the scintillating grid illusion (e.g., Matsuno & Sato, 2019; Qian et al., 2009; Schrauf et al., 1997). The stimuli consisted of small white disks, rectangles, or diamonds on a black square that subtended 18.7° × 18.7° of visual angle (Figure 2). The stimulus was presented on a white screen of the same color as the patches. The sizes (width and height) of the disks, rectangles, and diamonds were approximately 0.65°, 0.59°, and 0.82° of visual angle, respectively. The sizes of the shapes were determined in such a way that the area differences were minimized as much as possible under the constraints of pixelated images. The white patches were horizontally and vertically located at regular intervals. The center-to-center separation between patches was 1.18°, 1.77°, and 2.36° of visual angle in low, middle, and high stimulus density conditions, respectively. The number of patch elements was different among patch density conditions under the constraint of the size of a background black square. As for the patches, 7, 9, and 15 were placed in a horizontal or vertical line in low-, middle-, and high-density conditions, respectively. The luminance of the white patches and their black background were approximately 100 cd/m^2^ and 0 cd/m^2^, respectively.

#### Procedure

**Figure 3.**
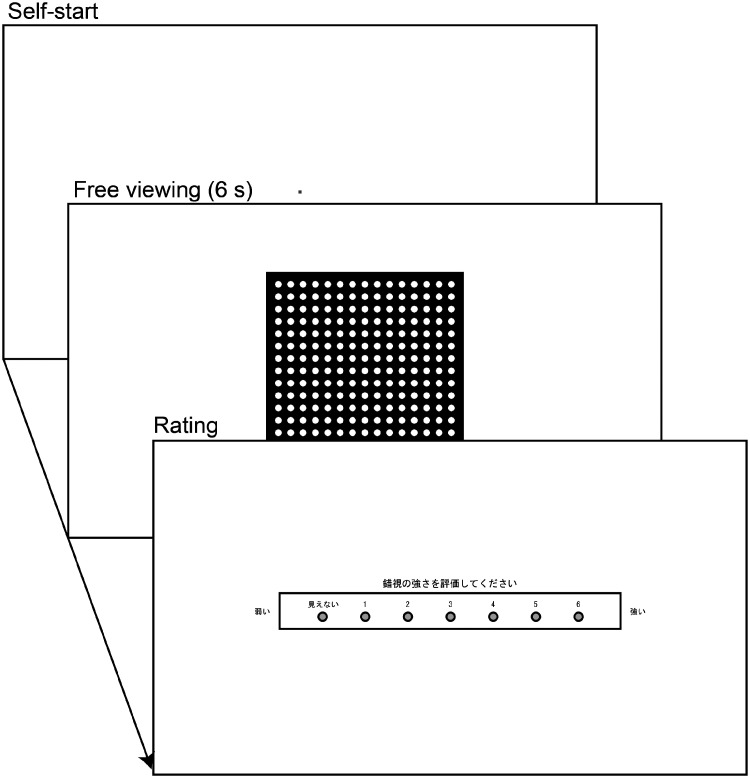
Schematic Diagram Illustrating a Trial in the Rating Task. The rating circles were presented with a title that instructed participants to *rate the strength of the illusion* with *weak* (left) and *strong* (right) notations in Japanese. The leftmost option was labeled as *invisible* in Japanese, and the other six options were numbered from 1 to 6.

The procedure of the task was designed based on previous studies on the scintillating grid illusion (Matsuno & Sato, 2019). At the start of a trial, a small red rectangle (0.30° × 0.30°) appeared at the center of the screen (Figure 3). When a participant made a mouse click, the rectangle was turned off, and a test stimulus was presented at the center of the screen. After a 6,000-ms interval, the stimulus disappeared, and a response scale and mouse cursor were presented at the center of the screen and below the scale, respectively. The response scale consisted of seven response circles to click and their labels. The leftmost label was an explicit description of *invisible*, instead of 0, in Japanese. The others were the numbers one to six, which showed the levels of visibility. A caption that instructed participants to rate the strength of the illusion and labels of *weak* and *strong* were added above, left, and right of the response circles, respectively. When the participant clicked their selection, the display turned blank, and a 3,000-ms intertrial interval was given. Participants were not required to fixate during the task, and no time limit was imposed on them for making responses.

Each observer participated in a practice session of 9 trials and a test session of 27 trials. The practice session, where each stimulus condition was tested once, was conducted before the test session. During a test session, nine trials, which contained one trial for each combination of the patch shape and density conditions, consisted of one block, and three blocks were repeated. The order of the trials was pseudo-randomized in a block.

Before the experiment, each participant was shown an example of the scintillating grid illusion with a white diamond patch and gray bars (Qian et al., 2009, Figure 2, left) printed on an A4-sized paper. They were instructed that their task was to observe the images similar to the figure but without gray lines; judge whether similar illusory black spots, even faintly, were observed; and select the options to rate the visibility of the illusion by a mouse click (see Supplemental Text for full instruction scripts). The participants were further informed that they were required to click the mouse to start a trial, gaze around the stimulus on the screen for 6 seconds, identify whether the illusory black spots in the white patches were visible or not, and rate the strength of the illusion by clicking an option displayed on the screen with labels of *invisible* or “1” to “6.” They were also given three additional instructions. One was that they were required to rate the visibility of the illusion based on the vividness, not the number, of the illusory spots. Second is that illusory spots could be absent in all trials, and in that case, there was no problem with selecting the *invisible* option in all trials. Third, other illusory phenomena than the black spots in the patches could be perceived, but those were not to be included in their rating judgments.

#### Data Analyses

The rating scores of the test trials were analyzed. The selection of the *invisible* option was treated as a rating of 0 in the analyses. The collected data were ordinal responses (rating values from 0 to 6) from each participant, and mixed ordinal probit regression models were therefore fitted for the analyses. The model included two stimulus conditions (patch shape and patch density) and their interaction as fixed factors and participants as a random factor. The statistical significance of the fixed effects was tested using the likelihood ratio test, and pseudo *R*^2^ statistics were calculated based on the likelihood ratio (Nagelkerke, 1991). For the significant main effects of the stimulus conditions, post hoc paired comparisons were performed with the Bonferroni correction. The paired comparisons were conducted based on the estimated marginal mean of the model predictions. All statistical analyses were conducted using R version 3.6 (R Core Team, 2019) and the emmeans (Lenth, 2019) and ordinal (Christensen, 2019) packages.

### Results and Discussion

**Figure 4.**
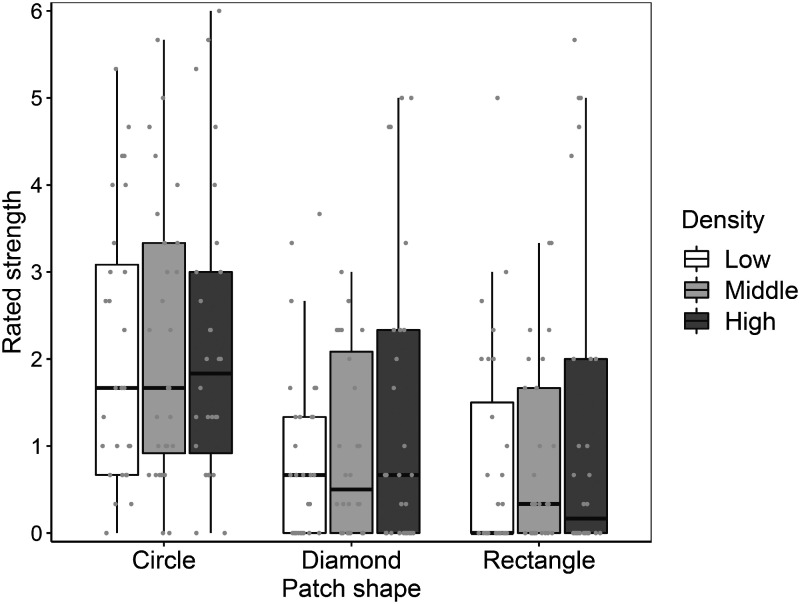
Results in Experiment 1. The left, middle, and right three boxes represent the distribution of the mean responses of participants in circular, diamond, and rectangle patch shape conditions, respectively. The box colors indicate different patch density conditions. Each gray dot is a mean rating score of a participant in each condition.

The illusion was experienced by the majority of the observers and was dependent on the contour shape of the patches (Figure 4). Only 1 among 28 participants consistently responded *invisible* in all of the conditions. In the comparison among the contour shape conditions, stimuli with circular patches induced more salient illusory spots than those with diamond and rectangle patches. On the other hand, the differences between density conditions seemed relatively small.

The results of the statistical testing confirmed these tendencies. The effect of the stimulus patch type was significant: χ^2^(2) = 157.16, *p* < .001, pseudo-*R*^2^= .20. Paired comparisons revealed that the rating score in the circular patch condition was significantly higher than the other two conditions (circle-diamond, *z* = 9.79, adjusted *p* < .001; circle-rectangle, *z* = 11.32, adjusted *p* < .001), while those in the diamond and rectangle conditions were not significantly different: *z* = 1.86, adjusted *p* = .188. The estimated means of the strength rating and their 95% confidence intervals were 2.01 [1.42, 2.60], 0.79 [0.43, 1.16], and 0.62 [0.31, 0.94] for the circle, diamond, and rectangle conditions, respectively. The effect of the stimulus density was also significant (χ^2^(2) =7.49, *p* = .024, pseudo-*R*^2^ = .01), although the effect on the rating scores was very small. The paired comparisons revealed that rating scores were significantly different between high- and low-density conditions (*z* = 3.19, adjusted *p* = .013), but not between the other possible comparisons (high-middle, *z* = 1.98; middle-low, *z* = 0.89; adjusted *p*s > .10). The estimated means of the strength ratings were 1.02 [0.63, 1.42], 1.11 [0.70, 1.52], and 1.30 [0.83, 1.76] for the low-, middle-, and high-density conditions, respectively. The interaction between the effects of patch shape and stimulus density was not significant: χ^2^(4) = 2.78, *p* = .60, pseudo-*R*^2^ = .00.

The advantage of the circular contour in seeing the illusion in comparison with diamond and rectangle contours is a replication of a previous study on the scintillating grid illusion (Qian et al., 2009). These results support the view that the shared mechanism underlies the illusions with and without grid bars. In addition, the visibility of the illusory spots was largely not influenced by the density of the visual elements, suggesting that crowding or local interference of the visual elements is not the cause of the illusory spots.

## Experiment 2

Experiment 2 examined the possible role of a square-lattice alignment of visual elements in generating illusory spots. For that purpose, the perceived strength of the illusion was compared between the stimuli with spatially aligned and misaligned visual elements.

### Method

Fourteen observers (seven females and seven males) ranging in age from 18 to 23 years (mean = 19.5, *SD* = 1.5) participated in Experiment 2. All had normal or corrected-to-normal visual acuity and were naïve as to the purpose of the experiments.

**Figure 5.**
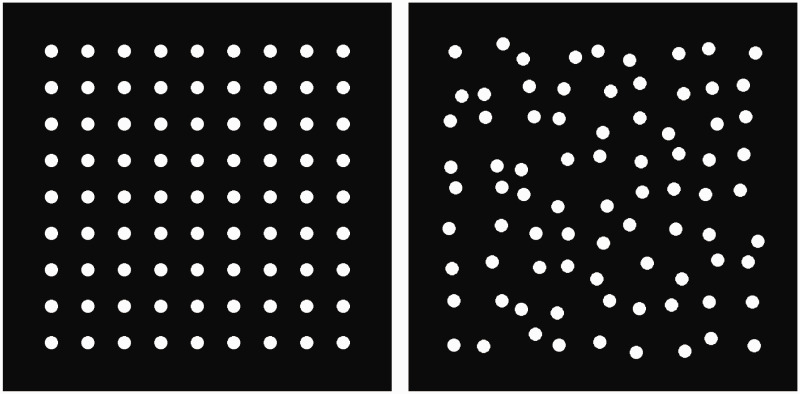
Stimuli Used in Experiment 2.

The stimuli, procedure, and data analyses were the same as in Experiment 1, except as reported here. The visibility of the stimuli in two conditions was compared (Figure 5). One was the circular patch stimulus used in Experiment 1. The patches were regularly aligned with a horizontal and vertical separation of 1.77° (middle-density condition in Experiment 1). The other stimulus was created by misaligning the patch locations of the stimulus in a trial-by-trial manner. The patches were pseudorandomly relocated in the range of −0.18° to 0.18° for each of the vertical and horizontal directions with a uniform distribution. Each observer participated in a practice session of two trials and a test session of eight trials. Each stimulus condition was tested once in a practice session and four times in a test session.

### Results and Discussion

**Figure 6.**
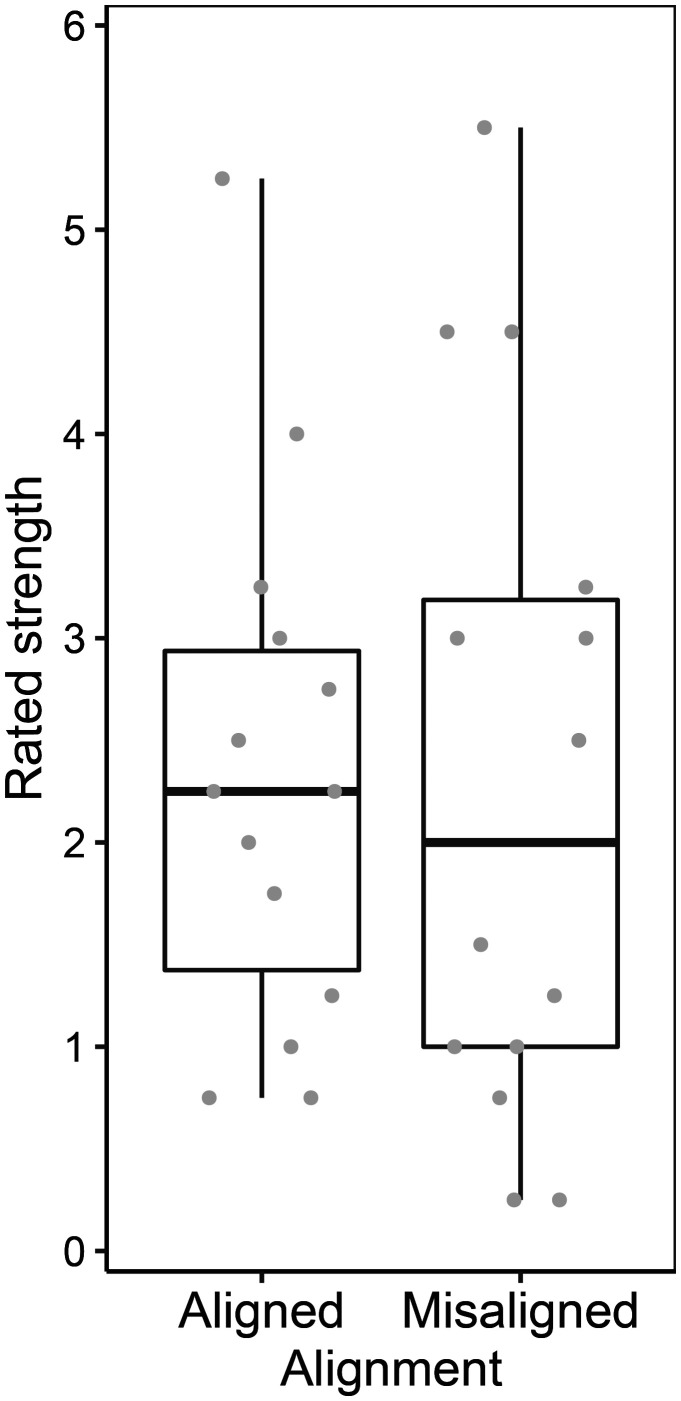
Results in Experiment 2. The left and right boxes represent the distribution of the mean responses of participants in aligned and misaligned conditions, respectively. Each gray dot is a mean rating score of a participant in each condition.

As shown in Figure 6, the rated strength of the illusory spots was not significantly different between the aligned and misaligned visual patterns: χ^2^(1) = 0.13, *p* = .72, pseudo-*R*^2^ = 0.00. The estimated mean rating scores and their 95% confidence intervals were largely overlapped between the aligned (2.39 [1.51, 3.28]) and misaligned (2.30 [1.41, 3.20]) conditions.

The illusion was not diminished at all with misaligned stimuli, suggesting that the global stimulus pattern is irrelevant to the generation of the illusory spots. This pattern of results was shared with the scintillating grid illusion. Qian et al. (2012; Experiment 3) revealed that the lateral offset of the patch locations did not alter the magnitude of the scintillating grid illusion.

These results also support the discussion on Experiment 1, showing that the local proximity of the elements largely does not affect the visibility of the illusion. In contrast to the aligned condition, the distance to the spatially nearest patch was shorter in the misaligned condition, while the number of elements was constant between the two conditions. If the local spatial interaction between visual elements is the cause of the illusion, the visibility of the illusion could be higher in the misaligned condition. Our results were not consistent with this assumption.

## General Discussion

The two experiments revealed that the illusory spots, like in the scintillating grid illusion, were perceived with a stimulus pattern without grid bars. The visibility of the illusion was modified depending on the shape of the white patches, and the properties of the shape dependency were common with the typical scintillating grid illusion with grid bars (Qian et al., 2009). The density and alignment of the visual elements largely did not affect the visibility of the illusion.

These results suggest that the primary cause of the scintillating grid illusion is independent of the visual processing of the grid bars. In addition, local spatial interaction between visual elements also would not fully explain our results. Therefore, we need to assume some other mechanism apart from the prior hypotheses that suppose that the grid bars are essential components.

On considering that the illusion is transiently perceived in our peripheral vision, the illusory spots could be induced solely depending on the limited visual processing of the patches. This explanation is consistent with the view that the scintillating grid illusion is caused by the interruption of the surface formation process of the white patches (Matsuno & Sato, 2019). Matsuno and Sato (2019) found that the size of the illusory spots of the scintillating grid illusion was determined by the size of the patch contour, not the width of the grid bars. In line with these findings, they noted that the percepts of the black illusory spots could be the lack of surface percepts of the patches caused by the interruption of the surface filling-in process with the spatial interference with the orientation processing of the grid bars. In light of the results of this study, the surface processing of the small visual elements could be temporarily incomplete in our peripheral vision just after eye movements, even without the interference with the grid bar processing. The grid bars in the scintillating grid illusion would play a role in enhancing this tendency.

According to this explanation, the absence or weakness of the illusory spots with diamond and rectangle contour patches could be due to the strength of the edge signals. It is known that the contours of the visual element play an important role in the surface formation process (Paradiso & Nakayama, 1991; van Lier et al., 2009) and that we are more sensitive to straight rather than curved contours (Pettet, 1999). The straight edge may segregate the area more definitely or rapidly and that may be advantageous for the surface perception in our peripheral visual processing, resulting in immunity to the illusion.

On the other hand, the discussions mentioned earlier that assumed the shared mechanism for the illusions with and without grid bars include two shortcomings to be carefully considered. First, the results that the illusory black spots are observed in the circular patterns were not consistent with a previous report, which claimed the scintillating grid illusion disappeared when the grid bars were curved (Geier et al., 2004; Geier & Hudák, 2017). One possible explanation would be that the contrast of the strength of the illusion when the original and deformed scintillating grid illusion was observed side-by-side or one after the other. When the observer expected the intensity of the original illusion, the presence of the much more sporadic and less salient illusory spots could be missed at a glance. In that case, careful and longer observation of the solely presented stimuli for a certain period (few seconds), as in our experimental procedure, could lead to noticing the presence of the weaker illusory spots. The other possibility would be that the addition of curvature itself may have an inhibitory effect on the illusion. Curvature is a salient visual feature that is important for visual object perception (e.g., Feldman & Singh, 2005). The curving of the grid could not merely be the loss of the straightness of the grid but the addition of the salient visual features to the stimulus, which could mask the weak illusory spots.

Second, the shared property of the appearance of the illusion, illusory black spots in circles, does not necessarily mean the shared underlying mechanism. Although the direct comparison between the illusion with and without grid bars was not conducted in this study, the illusion without grid bars reported here appears to be less salient, less frequent, and more transient. A possible reason for those differences is that the stimulus used in this study is under nonoptimal conditions. Alternatively, that may be because the illusion without grid bars is induced by a different unspecified mechanism from the illusion with grid bars.

In conclusion, the illusory black spots, as observed in the scintillating grid illusion, can be induced without grid bars. When we suppose that the illusion shared the mechanism with the original scintillating grid illusion, this observation indicates that the underlying mechanisms to induce the illusory spots of the scintillating grid illusion, and the illusory smudges of the Hermann grid illusion could be partly independent. In addition to the scintillating grid illusion, various phenotypes of illusion have been reported with combinations of simple grid bars and small visual elements (Kawabe et al., 2010; Ninio & Stevens, 2000; Qian & Mitsudo, 2016). Further investigations that untangle the shared and unique aspects of the series of grid illusions would help us to understand the basic properties of our visual processing.

## Supplemental Material

sj-pdf-1-ipe-10.1177_2041669520944418 - Supplemental material for Scintillating Grid Illusion Without the Grid Click here for additional data file. Supplemental material, sj-pdf-1-ipe-10.1177_2041669520944418 for Scintillating Grid Illusion Without the Grid by Toyomi Matsuno in i-Perception

sj-pdf-2-ipe-10.1177_2041669520944418 - Supplemental material for Scintillating Grid Illusion Without the Grid Click here for additional data file. Supplemental material, sj-pdf-2-ipe-10.1177_2041669520944418 for Scintillating Grid Illusion Without the Grid by Toyomi Matsuno in i-Perception
