# Supplementary material for: Scintillating Grid Illusion Without the Grid
Source: Iperception. 2020 Jul 23;11(4):2041669520944418. doi: 10.1177/2041669520944418 (PMC7383717; doi:10.1177/2041669520944418)
Supplement: sj-pdf-1-ipe-10.1177_2041669520944418 - Supplemental material for Scintillating Grid Illusion Without the Grid [file sj-pdf-1-ipe-10.1177_2041669520944418.pdf]

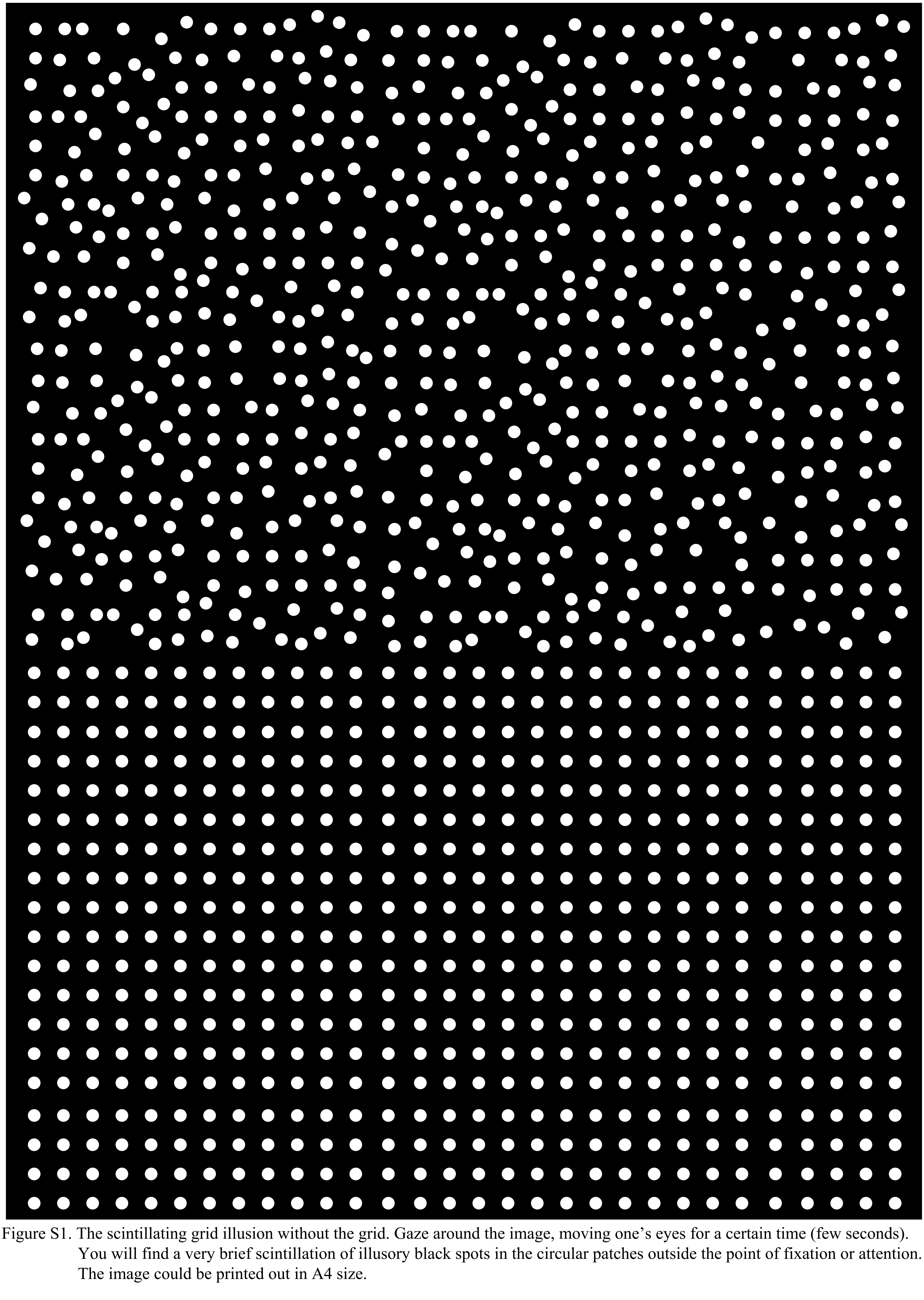

Figure S1. The scintillating grid illusion without the grid. Gaze around the image, moving one's eyes for a certain time (few seconds). You will find a very brief scintillation of illusory black spots in the circular patches outside the point of fixation or attention. The image could be printed out in A4 size.
