# Supplementary material for: Scintillating Grid Illusion Without the Grid
Source: Iperception. 2020 Jul 23;11(4):2041669520944418. doi: 10.1177/2041669520944418 (PMC7383717; doi:10.1177/2041669520944418)
Supplement: sj-pdf-2-ipe-10.1177_2041669520944418 - Supplemental material for Scintillating Grid Illusion Without the Grid [file sj-pdf-2-ipe-10.1177_2041669520944418.pdf]

Supplementary Text

Translated scripts of the instruction for the participants

In the experiment, you will be shown images similar to the figure (the scintillating grid stimulus with diamond patches and gray bars) but without gray lines. Your task is to observe the images; judge whether similar illusory black spots, even faintly, are observed; and select the options to rate the visibility of the illusion.

The options for the visibility rating are “invisible” or 1 to 6. Please select the “invisible” option when you do not perceive illusory spots at all, and “6” when you recognize the strong illusion.

You are required to rate the visibility of the illusion based on the vividness, not the number, of the illusory spots.

Other illusory phenomena apart from the black spots in the patches can be perceived, but those are not to be included in your rating judgments.

A flow of one trial was as follows.

1. Please click the mouse once to start a trial when the red rectangle appears at the center of the screen.

2. An image will be presented for six seconds. Please gaze around at the image on the screen, casting eyes on all the elements for six seconds, and identify whether the illusory black spots in the white patches are visible or not.

3. After six seconds, the options will appear. Please rate the strength of the illusion. When you do not perceive the illusion at all, there will be no problem in selecting the “invisible” option in all trials.”

4. You are allowed to have a break when the red rectangle is presented at the center of the screen.

You will receive a short practice session and a test session in succession.

Please ask me now if you have any questions.
